# PVT1: A Rising Star among Oncogenic Long Noncoding RNAs

**DOI:** 10.1155/2015/304208

**Published:** 2015-03-26

**Authors:** Teresa Colombo, Lorenzo Farina, Giuseppe Macino, Paola Paci

**Affiliations:** ^1^Institute for Computing Applications “Mauro Picone”, National Research Council, Via dei Taurini 19, 00185 Rome, Italy; ^2^Department of Computer, Control and Management Engineering, Sapienza University of Rome, Via Ariosto 25, 00185 Rome, Italy; ^3^Department of Cellular Biotechnologies and Hematology, Sapienza University of Rome, Viale Regina Elena 324, 00161 Rome, Italy; ^4^Institute for System Analysis and Computer Science “Antonio Ruberti”, National Research Council, Via dei Taurini 19, 00185 Rome, Italy

## Abstract

It is becoming increasingly clear that short and long noncoding RNAs critically participate in the regulation of cell growth, differentiation, and (mis)function. However, while the functional characterization of short non-coding RNAs has been reaching maturity, there is still a paucity of well characterized long noncoding RNAs, even though large studies in recent years are rapidly increasing the number of annotated ones. The long noncoding RNA PVT1 is encoded by a gene that has been long known since it resides in the well-known cancer risk region 8q24. However, a couple of accidental concurrent conditions have slowed down the study of this gene, that is, a preconception on the primacy of the protein-coding over noncoding RNAs and the prevalent interest in its neighbor MYC oncogene. Recent studies have brought PVT1 under the spotlight suggesting interesting models of functioning, such as competing endogenous RNA activity and regulation of protein stability of important oncogenes, primarily of the MYC oncogene. Despite some advancements in modelling the PVT1 role in cancer, there are many questions that remain unanswered concerning the precise molecular mechanisms underlying its functioning.

## 1. Introduction

Evidence strongly supports pervasive transcription of human genomes, even though only 2% of transcripts encode proteins [1–5]. Noncoding transcripts were for long time regarded as evolutionary junk or transcriptional noise, but, with the development of whole genome technologies, these noncoding RNAs (ncRNAs) are emerging as important regulators of gene expression. This variegated class of RNA species encompasses the well-known microRNAs (miRNAs), as well as the most recently acknowledged long noncoding RNAs (lncRNAs).

Noncoding transcripts are usually classified on the basis of their size [6]: miRNAs are single-stranded short RNAs (~22 nucleotides long) that posttranscriptionally regulate gene expression by translation inhibition or degradation of their target mRNAs [7–10]; long noncoding RNAs (lncRNAs) are broadly categorized as RNA longer than 200 nucleotides lacking extended open reading frames [11].

While miRNAs have been extensively studied [7–10], lncRNAs still constitute a new, potentially fascinating, territory yet to be explored. Recent studies have begun to associate subsets of lncRNAs to specific regulatory mechanisms [6, 12–16] of important biological processes, including cell proliferation, survival, differentiation, and chromatin remodelling both in* cis* and in* trans*. Many functional lncRNAs have been shown to play key roles in organ development and cancer; however the biological functions of the majority of lncRNAs largely remain to be identified. Increasing experimental evidence supports the hypothesis that lncRNAs may possess competing endogenous RNA (ceRNA) activity [6, 17–21]. This intriguing mechanism concerns the ability of some RNAs to compete for miRNA binding.

Our group recently developed a computational model able to identify putative ceRNAs among lncRNAs [21] and used it to build normal and cancer networks of miRNA-mediated sponge interactions (MMI-networks). The above ceRNA model, which we applied to breast cancer data from the Cancer Genome Atlas (TCGA) [22], allows characterization of lncRNAs in terms of oncogenes or tumor-suppressors protagonists in cancer. This speculation was gleaned by the observation of a marked rewiring in the ceRNA program between normal and pathological breast tissues. Most importantly, this analysis fueled our interest in the lncRNA PVT1 as being the first hub of the normal-MMI-network. We found that PVT1 favors binding to the crucial mir-200 family as the bone of contention with plenty of competitor mRNAs with declared importance in tumorigenesis since this miRNA family mediates over 80% of the interactions included in the PVT1 MMI-subnetwork in normal breast.

The human PVT1 gene (also known as Pvt1 oncogene) is a long intergenic noncoding RNA (lincRNA) homologous to the mouse plasmacytoma variant translocation gene (Pvt1). Indeed this locus has been first discovered in the mid 80s in mouse as frequently involved in a variant translocation in plasmacytomas [23, 24]. Shortly after, the PVT1 locus emerged as a site of variant translocations also in human Burkitt's lymphomas [25–27]. Around the same years, systematic studies conducted in mice and rats to find common retroviral integration sites in T lymphomas revealed the Pvt1 gene as one such locus, suggesting its oncogenic potency [28–30]. This oncogenic attitude of PVT1 gained support from later evidences of frequent upregulation [21, 31–35] and amplification in a wide variety of cancers. Moreover, PVT1 lies in a recognized cancer risk locus that it shares with the well-known MYC oncogene. This site, which maps to chromosome 8 in human, is among the top target of copy number alterations in cancer and has equivalents in both mouse (chromosome 15) and rat (chromosome 7) [25, 28, 36, 37].

In the light of all the above, we can hazard a guess that if PVT1 was a protein-coding gene it would have been at the heart of countless studies aimed at the functional characterization since its discovery. Rather, being a noncoding transcript has slowed the process of shading light on its mechanisms of action, for reasons of both technical and cultural nature. In the early years of its discovery, in fact, on the one hand the classical molecular techniques for functional investigation were not suited to detecting and analyzing noncoding RNAs; on the other hand it was hardly conceivable that what was still deemed as transcriptional noise could play an active role in the regulation of important processes.

**Figure 1:**
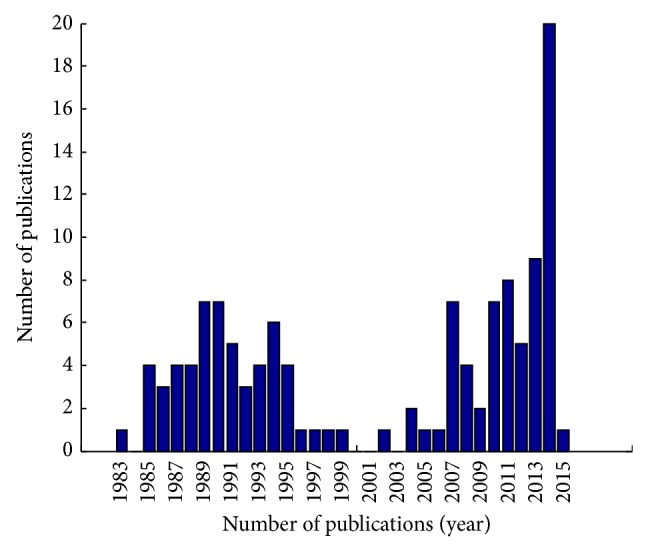
Rising interest in the oncogenic lncRNA PVT1. Barplot showing the frequency for publications having the word “PVT1” in the title and/or abstract listed in PubMed http://www.ncbi.nlm.nih.gov/pubmed, as of January 2015.

Instead, the technological revolution of recent years along with the unprecedented appreciation of the multifaceted world of noncoding RNAs has led to a renewed interest in the investigation of the PVT1 function. This growing interest is reflected in the multitude of papers published in the last few years on PVT1 (Figure 1). As a consequence, new interesting hypotheses are emerging on the regulatory networks involving PVT1 in normal cells and disease. Nonetheless, the precise mechanism of PVT1 functioning still remains largely unknown.

## 2. PVT1 Genomic Locus

**Figure 2:**
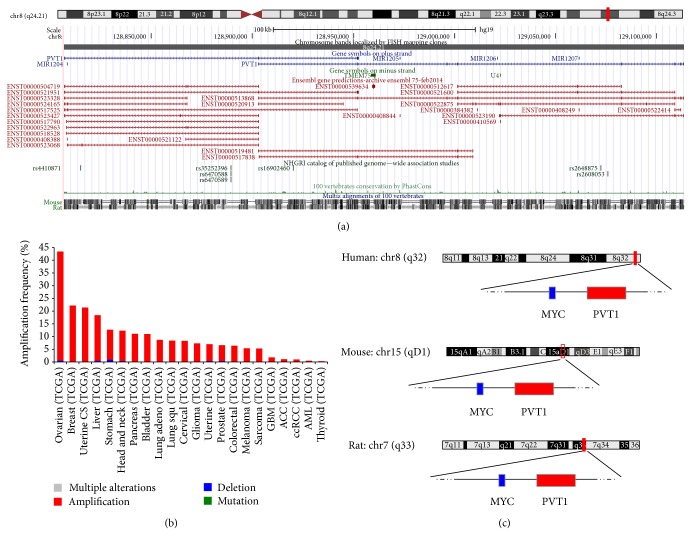
The PVT1 locus in humans. (a) UCSC Genome Browser (http://genome.ucsc.edu/) view of the 8q24.21 region in humans, which contains the PVT1 gene. (b) Barplot of cross cancer alteration frequency for the PVT1 gene. *x*-axis: different cancer types from the Cancer Genome Atlas [22]. *y*-axis: total frequency of PVT1 gene alterations. Summary of TCGA cross cancer data and visualization obtained from the cBioPortal for Cancer Genomics (http://www.cbioportal.org/). (c) Sketch of rat and mouse genomic regions syntenic to the human MYC/PVT1 region.

In humans the chromosomal locus encoding PVT1 (Figure 2(a)) maps to band 4 of region 2 on the long arm of chromosome 8 (8q24), specifically to sub-subband 1 of subband 2 therein (8q24.21). The 8q24 locus has often attracted the attention of researchers for at least three main reasons: (i) it constitutes a preferred site for genetic aberrations, including translocation [25, 37, 38], amplification [25, 38–41], and viral integration [42, 43] in a wide variety of cancers and other malignancies (Figure 2(b)); (ii) it encompasses multiple risk loci for different diseases [44–56]; and (iii) it includes a 1.8-megabases region of* gene desert*, so termed due to paucity of transcriptional activity [57, 58], which is considered a cancer risk site likely involved in long-range interactions that affect the expression of distant genes [45, 59, 60]. Indeed, chromosome conformation capture- (3C-) based studies have confirmed extensive physical contacts of this risk locus with multiple genomic regions, including intrachromosomal interactions with the PVT1 locus [60, 61].

Another remarkable feature of the PVT1 locus is to reside some 60 kilobases (kb) 3-prime of the well-known MYC oncogene and different functional interactions between these two genes have been proposed [41, 62]. Adding to this, the MYC-PVT1 pair of genes results syntenic in human (chromosome 8), mouse (chromosome 15), and rat (chromosome 7) (Figure 2(c)) [25, 37]. Yet, there is also evidence for independent contribution of PVT1 and MYC to disease [31, 60].

Both the mouse and the rat homologous of human PVT1 (Pvt1 and Mis-1, resp.) constitute sites of frequent retroviral integration [59, 63–66] and, similarly to the human counterpart, sites of frequent translocations in B-cell derived tumors (i.e., Burkitts lymphoma, plasmacytoma, and immunocytoma in human, mouse, and rat, resp.) [23, 25, 67].

## 3. PVT1 Expression in Normal Tissues and in Disease

**Figure 3:**
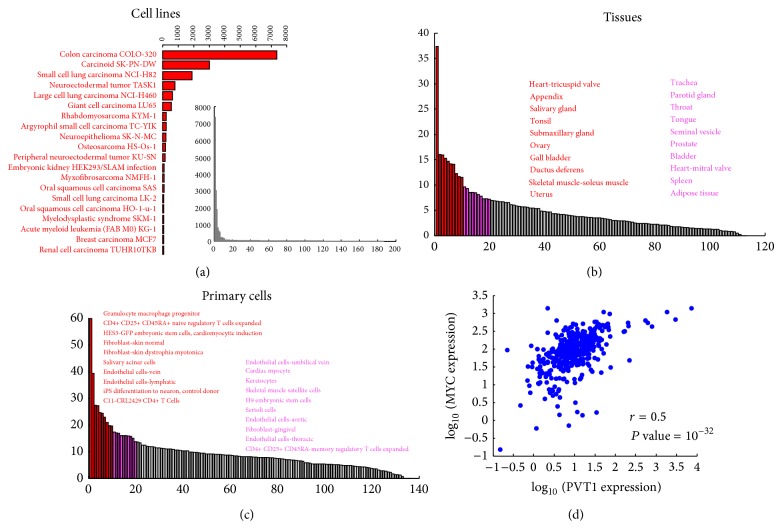
The PVT1 expression in a human atlas. (a–c) Barplots of sorted PVT1 expression values across human cell lines, tissues, and primary cells, respectively. Expression data are taken from the atlas recently released by the FANTOM consortium [71]. *y*-axis: normalized expression value (tags per million). *x*-axis: human cell lines, tissues, and primary cells, respectively. In each panel, the name of the top 20 samples is listed and the corresponding bars are similarly colored. In (a), the inset shows the overall sorted PVT1 expression values in human cell lines. The PVT1 expression data plotted in (a–c) (i.e., 186 cell lines, 113 tissues, and 136 primary cells) constitute a subset of the full FANTOM atlas (>850 samples) that was selected to reduce redundancy in the plots. (d) Scatterplot of the MYC versus PVT1 expression values across all the samples analyzed in (a–c). The Pearson correlation (*r*) and the corresponding *P* value are shown.

**Figure 4:**
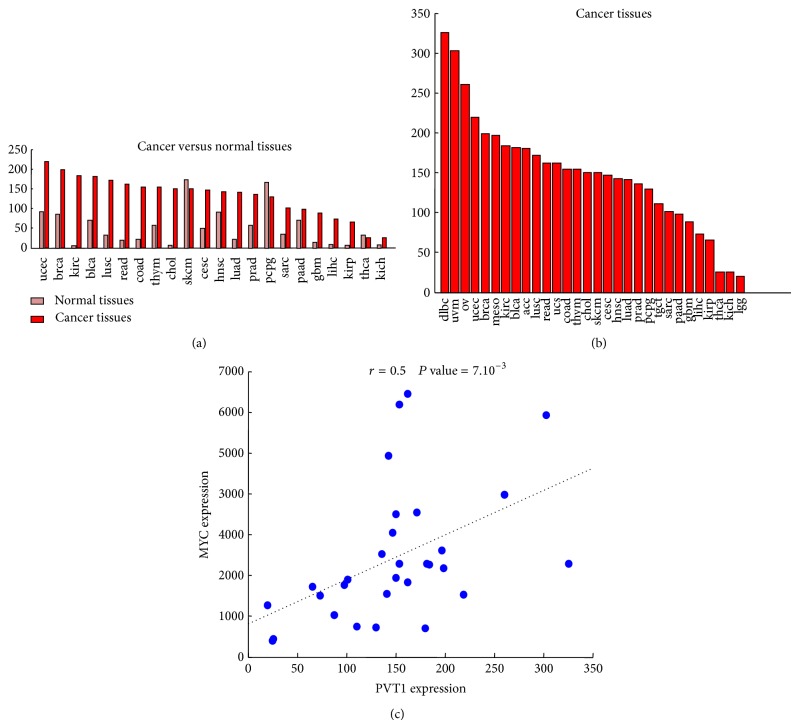
PVT1 expression in cancer. (a) Barplots of mean PVT1 expression in cancer versus normal tissues from the Cancer Genome Atlas [22]. Values are ordered by decreasing abundance in cancer. *y*-axis: normalized expression value (FPKM). *x*-axis: TCGA tissues. (b) Barplots of mean PVT1 expression in TCGA cancer tissues, ordered by decreasing abundance. *x*-axis and *y*-axis: same as in (a). (c) Scatterplot of the MYC versus PVT1 expression values across all the samples analyzed in (a-b). The Pearson correlation (*r*) and the corresponding *P* value are shown. Abbreviations: acc: adrenocortical carcinoma; blca: bladder urothelial carcinoma; lgg: brain lower grade glioma; brca: breast invasive carcinoma; cesc: cervical squamous cell carcinoma and endocervical adenocarcinoma; chol: cholangiocarcinoma; coad: colon adenocarcinoma; gbm: glioblastoma multiforme; hnsc: head and neck squamous cell carcinoma; kich: kidney chromophobe; kirc: kidney renal clear cell carcinoma; kirp: kidney renal papillary cell carcinoma; lihc: liver hepatocellular carcinoma; luad: lung adenocarcinoma; lusc: lung squamous cell carcinoma; dlbc: lymphoid neoplasm diffuse large B-cell lymphoma; meso: mesothelioma; ov: ovarian serous cystadenocarcinoma; paad: pancreatic adenocarcinoma; pcpg: pheochromocytoma and paraganglioma; prad: prostate adenocarcinoma; read: rectum adenocarcinoma; sarc: sarcoma; skcm: skin cutaneous melanoma; tgct: testicular germ cell tumors; thym: thymoma; thca: thyroid carcinoma; ucs: uterine carcinosarcoma; ucec: uterine corpus endometrial carcinoma; uvm: uveal melanoma.

The PVT1 gene [68] spans across a genome interval of over 300 kb (i.e., bases 128806779–129113499 within the February 2009 human genome build GRCh37/hg19) on the forward strand of chromosome 8. The high complexity of the gene architecture of PVT1 has been long known [69] and most recent studies based on high-throughput techniques for gene expression analysis have confirmed it [21, 31, 41]. In fact, the large PVT1 locus gives rise to over 20 different variants of the lncRNA according to the Ensembl annotations of the human genome (release 75) [70] (Figure 2(a)). Moreover, a landmark study recently published by the FANTOM consortium [71] that compiled a whole genome map of human and mouse promoters by using cap analysis of gene expression (CAGE) technique has annotated multiple transcription start sites (TSS) overlapping the PVT1 locus. The same PVT1 locus also produces a cluster of six annotated microRNAs (namely, miR-1204, miR-1205, miR-1206, miR-1207-5p, miR-1207-3p, and miR-1208) [72]. The FANTOM study also provided the first comprehensive atlas of gene expression across human body and cell lines from which a global view of the expression pattern of PVT1 can be derived (Figures 3(a)–3(c)). Genome-wide expression studies in humans and other organisms have reported that neighboring genes tend to have correlated expression patterns [73–75]. Indeed, a comparison of the expression levels of PVT1 versus its neighbor MYC gene across the samples included in the above compendium of gene expression data showed a statistically significant Pearson correlation (*r* = 0.5 and *P* values = 10^−32^, Figure 3(d)). This coexpression could be indicative of a transcriptional coregulation and of a possible involvement in common functional pathways. Overall, the expression levels of PVT1 appear to be higher (up to 100 times) in cancer cell lines than in human primary cells and tissues. This trend strengthens the experimental evidences of its overexpression in a wide variety of tumors [31–35], with or without a concurrent copy number amplification [31]. The increased expression in tumors (Figures 4(a)-4(b)) and the high Pearson correlation between PVT1 and MYC expression levels (Figure 4(c)) are both fully recovered in an analysis of the large collection of tumor expression data provided by TCGA project.

## 4. PVT1 Function

**Figure 5:**
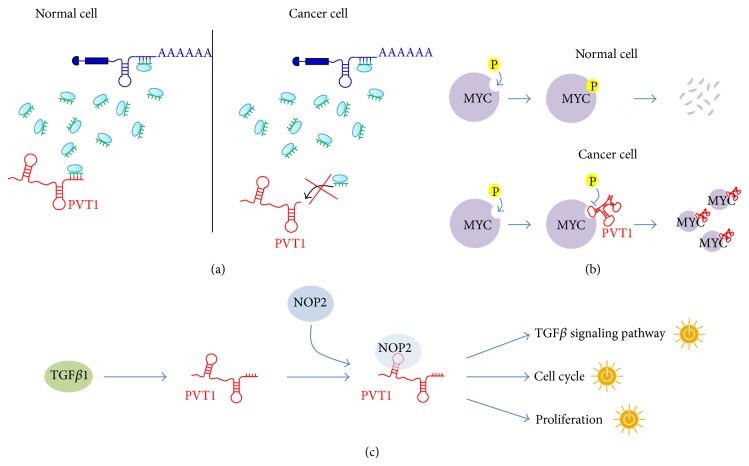
The PVT1 functions. (a) Model of PVT1 ceRNA mechanism operating in normal breast but not in breast cancer tissues possibly due to a prevalence in cancer of expression of PVT1 isoforms lacking recognition elements for the miRNA to be sponged, as it has been hypothesized for the case of mir-200 family based on sequence analysis of alternative PVT1 isoforms [21]. (b) Model of PVT1 positive regulation of the MYC protein stability by rescuing MYC from phosphorylation [87]. This figure is inspired by [87]. (c) The proposed model of TGF*β*1/PVT1/NOP2 regulatory network [41]: TGF*β*1 promotes the interaction between PVT1 and NOP2 that in turn activates proliferation, cell cycle, and TGF*β* signalling pathways. The sketches of yellow switches in the figure symbolize the pathway activation.

By analysing the breast cancer data set provided by TCGA, we found [21] that PVT1 acts as ceRNA in the normal-MMI-network but not in cancer (Figure 5(a)). Moreover, PVT1 revealed a net binding preference towards the mir-200 family, which it antagonizes to regulate the expression of hundreds of mRNAs in the normal case. In fact in normal breast we reported for PVT1 a subnetwork of ceRNA interactions connecting it to 753 different mRNAs (i.e., 50% of total mRNAs comprising the whole normal-MMI-network) and surprisingly over 80% of these ceRNA interactions stood from competition for binding with members of the same miRNA family: the mir-200 family.

In terms of topological properties, PVT1 switches from being the first of the hubs in the normal-MMI-network to fall outside the list of nodes of the cancer network. Interestingly, recent studies suggested a role for PVT1 in the pathophysiology of breast cancer by virtue of PVT1-mediated inhibition of apoptosis and increasing of proliferation, when overexpressed [31].

Among competitors of PVT1 for binding to the mir-200 family in normal breast tissues, there are some important regulators of breast tissue morphogenesis and development, such as CDH1; all the three members of the extended p53 family (i.e., TP53, TP63, and TP73); two members of the mammalian RUNX family (i.e., RUNX1 and RUNX3); and GATA3. CDH1 (also known as E-cadherin) is a crucial cell-cell adhesion molecule considered as an epithelial cell marker. Loss of function of CDH1 has been associated with increased proliferation, invasiveness, and/or metastasis in several epithelial cancers, including breast cancer [76]. TP53 is the most extensively studied tumor suppressor and acts in response to diverse forms of cellular stresses to induce cell cycle arrest, apoptosis, and senescence [77, 78]. The two identified homologues, TP63 and TP73, have also been related to apoptosis, and a possible role as tumor suppressors has been suggested [77]. RUNX1 is the predominant RUNX family member expressed in human breast epithelial cells and there is a growing body of evidence suggesting its possible role as a breast cancer suppressor [79–82]. RUNX3 has been recently reviewed as a tumor suppressor, specifically in human breast cancer, with decreasing expression associated with disease progression [80, 83]. Finally, GATA3 has been linked to mammary gland morphogenesis, mammary tumor differentiation, and metastasis [84].

Thus, the PVT1 neighborhood in the normal-MMI-network encompasses cancer related genes as well as genes involved in mammary gland development and cell morphogenesis and the sponge program orchestrated by PVT1 results completely abolished in cancer. Taken together these findings may be indicative of a possible PVT1 surveillance role aimed to preserve cell-cell adhesion. Indeed, mammary gland morphogenesis results from the coordination of diverse cellular processes involving cell-cell adhesion, migration, proliferation, and apoptosis. Thus, PVT1 controlling circuit may provide further insight in solving this complicated puzzle.

The specific conditions required for ceRNA network to occur are still far from being determined. The importance of the relative concentration of the ceRNAs, as well as their related miRNAs, has been recently emphasized [85]. Large changes in the ceRNA expression levels either overcome or relieve the miRNA repression on competing ceRNAs; similarly, a very large miRNA overexpression may abolish competition. However, both the mir-200 family members and PVT1 appear highly upregulated in the breast cancer dataset analysed in [21]. Thus, we formulated an alternative hypothesis for the abolishment of the ceRNA activity of PVT1 in cancer, prompted by the existence of multiple isoforms produced by its genomic locus. In fact, despite most of the PVT1 alternative isoforms harboring seed matches for the mir-200 family members, two isoforms lack the putative binding sites for this miRNA family. Hence, the observed withdrawal in cancer of the PVT1 sponge activity may be due to the preferential expression of these two isoforms, independently from the abundance of PVT1 (Figure 5(a)).

A completely different way of functioning of PVT1 comes from new findings that highlight its involvement in the regulation of the oncoprotein MYC [41]. According to this study, PVT1 controls levels of MYC through regulation of the protein stability and they both cooperate to promote cell proliferation in cancer. In [41], the authors analyzed copy number variation data from two large cancer databases (i.e., Progenetix and TCGA) and consistently observed (98% of cases) a cogain of MYC and PVT1 across a wide variety of cancers with amplified 8q24 region. This PVT1 and MYC protein coamplification was confirmed in a panel of 8 human primary tumors. Moreover, depletion of PVT1 by small interference RNAs (siRNAs) caused the reduction of MYC protein levels in a human colorectal cancer cell line and nuclear colocalization of PVT1 and MYC was also shown. In a breast cancer mouse model, Tseng and coworkers observed a halving in the proliferation upon either MYC or PVT1 knockdown. The observed proliferative phenotype confirmed what was already reported in a previous study conducted in ovarian and breast cancer cell lines [31]. Interestingly, while the inhibition of either PVT1 or MYC affected proliferation, a proapoptotic phenotype was exclusively observed when silencing PVT1 [31]. All the above pieces of information were reconciled by Tseng and coworkers in a unified model where the lncRNA PVT1 interacts with MYC in the nucleus, either directly or indirectly, and protects the MYC protein from degradation by reducing phosphorylation of its threonine 58 residue (Figure 5(b)).

A recent screening for liver oncofetal (i.e., fetal specific molecules misexpressed in cancer) lncRNAs in a mouse model for hepatocellular carcinoma (HCC) supported the role for PVT1 in regulating proliferation and the same phenotype was confirmed in human HCC cell lines [35]. In line with previous reports in other epithelial cancers [31, 32, 34, 35], PVT1 was found to be upregulated in cancer compared to noncancerous tissues and this upregulation was associated with a worse prognosis in a panel of HCC patients. Moreover, the authors found that the upregulated PVT1 induces cell cycle genes and activation of the TGF*β*1 signaling pathway (Figure 5(c)) and suggested a possible feedback loop between this lincRNA and TGF*β*1 [35]. Interestingly, the same authors found that PVT1 interacts with the proliferation-associated nucleolar protein NOP2 and stabilizes it from degradation [35]. Remarkably, this model of functional interaction is similar to that proposed between PVT1 and MYC [41].

## 5. Conclusions

A wide range of solid tumors often carry amplification of the 8q24 locus, a large genomic region spanning the PVT1 gene and its bulky neighboring MYC. Despite early recognition of the importance of this locus with respect to many malignancies, the well-known MYC oncogene has repeatedly been considered the first guilt by association. While the role of MYC in cancer has been proved beyond any doubt, the presence of this famous neighbor together with the enigmatic noncoding nature of PVT1 has hampered prompt recognition that another important oncogene coexisted with MYC in the 8q24 risk region. Nowadays, the accumulating evidences showing that PVT1 and MYC have separate and distinct oncogenic potential [21, 31, 32, 86] caused PVT1 to acquire independent individuality with respect to MYC.

In conclusion, the foregoing considerations suggest to think that the time has come for a thorough investigation of the role played by PVT1 in tumors, a challenge full of promise.
